# Implementation of second-tier tests in newborn screening for the detection of vitamin B_12_ related acquired and genetic disorders: results on 258,637 newborns

**DOI:** 10.1186/s13023-021-01784-7

**Published:** 2021-04-30

**Authors:** Sonia Pajares, Jose Antonio Arranz, Aida Ormazabal, Mireia Del Toro, Ángeles García-Cazorla, Aleix Navarro-Sastre, Rosa María López, Silvia María Meavilla, Mariela Mercedes de los Santos, Camila García-Volpe, Jose Manuel González de Aledo-Castillo, Ana Argudo, Jose Luís Marín, Clara Carnicer, Rafael Artuch, Frederic Tort, Laura Gort, Rosa Fernández, Judit García-Villoria, Antonia Ribes

**Affiliations:** 1 Sección de Errores Congénitos del Metabolismo-IBC, Servicio de Bioquímica Y Genética Molecular, Hospital Clínic de Barcelona, C/ Mejía Lequerica S/N, Edificio Helios III, 08028 Barcelona, Spain; 2 Center for Biomedical Research Network on Rare Diseases (CIBERER), Madrid, Spain; 3 Unit of Metabolic Diseases, Hospital Vall D’Hebrón, Barcelona, Spain; 4 Inborn Errors of Metabolism Unit, Hospital Sant Joan de Déu, Barcelona, Spain; 5 Biomedical Research Institute, August Pi I Sunyer (IDIBAPS), Barcelona, Spain; 6 Maternal and Child Health Service, Public Health Agency of Catalonia, Health Department, Government of Catalonia, Barcelona, Spain

**Keywords:** Vitamin B_12_ deficiency, Homocysteine, Methylmalonic acid, Methylcitric acid, Newborn screening, Second-tier test, Methylmalonic acidemia, Propionic acidemia, Homocystinuria

## Abstract

**Background:**

Alteration of vitamin B_12_ metabolism can be genetic or acquired, and can result in anemia, failure to thrive, developmental regression and even irreversible neurologic damage. Therefore, early diagnosis and intervention is critical. Most of the neonatal cases with acquired vitamin B_12_ deficiency have been detected by clinical symptoms and only few of them trough NBS programs. We aim to assess the usefulness of the second-tier test: methylmalonic acid (MMA), methylcitric acid (MCA) and homocysteine (Hcys) in our newborn screening program and explore the implications on the detection of cobalamin (vitamin B_12_) related disorders, both genetic and acquired conditions.

**Methods:**

A screening strategy using the usual primary markers followed by the analysis of MMA, MCA and Hcys as second tier-test in the first dried blood spot (DBS) was developed and evaluated.

**Results:**

During the period 2015–2018 a total of 258,637 newborns were screened resulting in 130 newborns with acquired vitamin B_12_ deficiency (incidence 1:1989), 19 with genetic disorders (incidence 1:13,613) and 13 were false positive. No false negatives were notified. Concerning the second-tier test, the percentage of cases with MMA above the cut-off levels, both for genetic and acquired conditions was very similar (58% and 60%, respectively). Interestingly, the percentage of cases with increased levels of Hcys was higher in acquired conditions than in genetic disorders (87% and 47%, respectively). In contrast, MCA was high only in 5% of the acquired conditions versus in 53% of the genetic disorders, and it was always very high in all patients with propionic acidemia.

**Conclusions:**

When screening for methylmalonic acidemia and homocystinuria, differential diagnosis with acquired vitamin B_12_ deficiency should be done. The results of our strategy support the inclusion of this acquired condition in the NBS programs, as it is easily detectable and allows the adoption of corrective measures to avoid the consequences of its deficiency.

**Supplementary Information:**

The online version contains supplementary material available at 10.1186/s13023-021-01784-7.

## Background

Methylmalonic acidemia, propionic acidemia and homocystinuria, including remethylation defects, are an extensive group of inherited genetic defects included in the expanded newborn screening (NBS) programs in several countries[1, 2].

Methylmalonic acid (MMA), methylcitric acid (MCA) and homocysteine (Hcys) are widely known biomarkers of genetic conditions leading isolated or combined methylmalonic acidemia and homocystinuria [3, 4], or propionic acidemia [5]. However, elevations of MMA, Hcys and MCA can also be the result of secondary alterations, such as acquired vitamin B_12_ (cobalamin) deficiency [6]. Cobalamin functions in two coenzyme forms: adenosylcobalamine (AdoCbl), which acts as coenzyme in the conversion of methylmalonyl–CoA to succinyl–CoA through methylmalonyl-CoA mutase (EC 5.4.99.2), and methylcobalamine (MeCbl), which acts as coenzyme of methionine synthase (EC 2.1.1.13) in the conversion of Hcys to methionine (Met) with methyltetrahydrofolate, the other essential cofactor of this reaction, converted to tetrahydrofolate in the process [3]. Vitamin B_12_ must be taken from the diet, particularly from meat, eggs, fish, and milk. Consequently, any alteration of vitamin B_12_ metabolism, as well as alterations of its absorption, transport, or low intake could cause high levels of MMA and/or Hcys and even MCA accumulation and low levels of Met.

Methylmalonic acidemia and homocystinuria, independently of its origin, can result in anemia, failure to thrive, developmental regression and even irreversible neurologic damage if the deficiency is prolonged [7–13]. Therefore, early diagnosis and intervention is critical. NBS detection is performed through the analysis of propionylcarnitine (C3), Met and the ratios C3/acetylcarnitine (C3/C2) and C3/Met in dried blood spots (DBS). Recently, heptadecanoylcarnitine (C17) has been proposed as new biomarker [14]. However, due to the high rate of false positives using these markers alone, the analysis of second-tier test is required [15–17]. Despite the great contribution of NBS programs to early identification and treatment of these conditions [18–22], most of the neonatal cases with acquired vitamin B_12_ deficiency have been detected by clinical symptoms and only few of them trough NBS programs. Recently reported results of a NBS pilot study recommended the inclusion of acquired vitamin B_12_ deficiency in the NBS programs [23, 24].

We aim to assess the usefulness of the second-tier test: MMA, MCA and Hcys in our newborn screening program and explore the implications on the detection of genetic and acquired conditions of vitamin B_12_ deficiency.

## Materials and methods

Subjects, aminoacids and acylcarnitines analysis, as well as organic acid analysis on dried urine spots (DUS) are described in Additional file 1.

### Analysis of MMA, MCA, and Hcys on dried blood spots (DBS)

Isotopically labeled MMA and Hcys (MMA-_d3_ and Hcys-_d8_, respectively) were purchased from Cambridge Isotope Laboratories (Tewksbury, MA, USA). MCA and MCA-_d3_ were provided by CDN Isotopes (Pointe-Claire, Quebec, Canada). MMA, Hcys, formic acid and DL-dithiothreitol (DTT) were obtained from Sigma-Aldrich (Saint Louis, USA). Methanol and acetonitrile (LC/MS PAI grade) were obtained from EMD Millipore Corporation (Madrid, Spain). Milli-Q system (Millipore) purified water was used.

#### Sample preparation

A 4.7 mm disc was punched from each DBS and transferred to a 96-well plate; 175 μL of a solution containing a mix of isotopically labeled standards (0.5 μmol/L Hcys-_d8_, 1 μmol/L MMA-_d3_, and, 1 μmol/L MCA-_d3_), and DTT (42 mmol/L), in Milli-Q water with 0.2% formic acid, were added to each well. The plate was capped and placed into a shaker. Samples were rotated at 1350 rpm for 1 h and then transferred to a clean 96-well filter plate and centrifuged at 4000 rpm for 40 min; 30 μL of the filtered sample were transferred to a clean 96-well round bottom plate, then 120 μL of Milli-Q water containing 0.2% formic acid were added to each well. Once the plate was capped it was ready to inject. A calibration curve of 6 points with spiked concentrations of 0, 5, 10, 20, 50 and 100 μmol/L of Hcys and, 0, 2.5, 5, 12.5, 25 and 50 μmol/L of MMA and MCA on DBS was used for quantification. DBS quality control samples at three concentration levels for each metabolite were analyzed within each batch of samples: 5, 12.5 and 25 μmol/L for Hcys, and 2.5, 6.25 and 12.5 μmol/L for MMA and MCA, respectively.

#### UPLC-tandem mass spectrometry conditions

MMA, MCA and Hcys on DBS were analyzed by an ultra performance liquid chromatography-tandem mass spectrometry, (UPLC-Xevo TQS Waters, Milford, MA, USA). The chromatographic separation was performed on an Acquity I-Class UPLC with BEH C18 column (2.1 mm x100mm, 1.7 μm, Waters, Milford, MA, USA). The mobile phase consisted of 0.2% formic acid in water (mobile phase A) and 0.2% formic acid in methanol:water (95:5) (mobile phase B). An isocratic elution at 60ºC with 95% mobile phase A (0.2% formic acid in water) and 5% mobile phase B (0.2% formic acid in methanol:water; 95:5) using a flow rate of 0.5 mL/min during 1.5 min was established. The injection volume was 10 μL.

Mass spectrometer was operated in the electrospray positive ion mode for Hcys and Hcys-_d8_, and negative ion mode for MMA, and MMA-_d3_, MCA and, MCA-_d3_, using multiple reaction monitoring (MRM) mode. Monitored transitions for unlabelled and labeled compounds were: MMA *m*/*z* 117 > 73 and *m*/*z* 120 > 76; Hcys *m*/*z* 136 > 90 and *m*/*z* 140 > 94; MCA *m*/*z* 205 > 125 and *m*/*z* 208 > 128. Nitrogen and argon were used as nebulizing and collision gas, respectively. Dwell time for each transition was 60 ms and the inter-channel delay was 20 ms. Run time was 1.5 min. The following instrumental setting was used: source temperature 150 °C, desolvatation temperature 550 °C and capillary voltage 3 kV. Data acquisition and data analysis were performed using MassLynxTM (V3.2) software. The quantification of the compounds was relative to their corresponding internal standards. External calibration curves were used.

#### Method validation

The method was validated for linearity, limit of detection, limit of quantification, imprecision, accuracy and recovery. Within-day imprecision, between-day imprecision, accuracy and recovery were evaluated by the analysis of quality control samples at three concentration levels: 3, 15 and 40 μmol/L of MMA; 6, 30 and 80 μmol/L of Hcys; and 3, 15 and 40 μmol/L of MCA. The accuracy was expressed as percentage of relative error (results reported in Additional file 2).

### Screening approach to the diagnosis

At present our screening panel by tandem MS/MS consists of 20 inherited metabolic (Additional file 3). When screening for propionic acidemia or methylmalonic acidemia and homocystinuria, second-tier tests in the first DBS are necessary to avoid false positive or false negative results. Nonetheless, from 2013 to 2014, second-tier test analysis was not available at our laboratory. Therefore, when primary markers were altered our strategy was to ask for a second DBS and a DUS. DBS was used to reanalyze the primary markers and DUS to analyze organic acids.

**Fig. 1:**
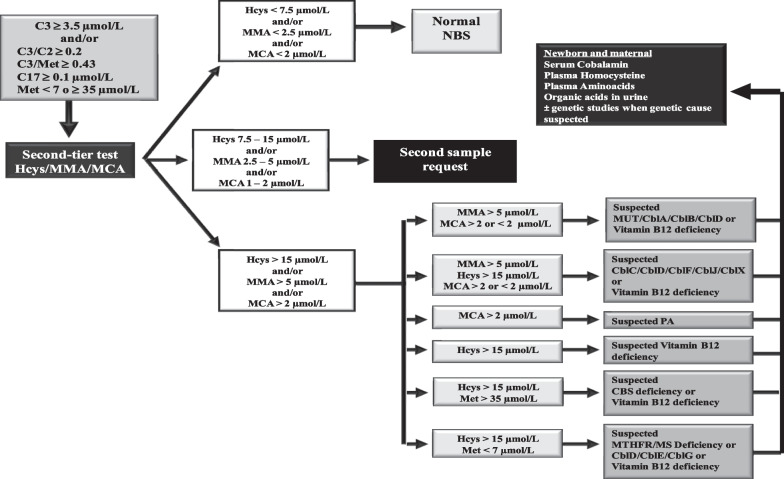
Algorithm for the detection of genetic disorders and acquired vitamin B_12_, from 2015 to nowadays. Cut-off values in μmol/L: C3: initial strategy: < 4.5 (99th ptl); current strategy: 3.5 (96th ptl); Met: 7–35 (1th ptl and 99.7th ptl, respectively); C17: < 0.1 (99.9th ptl); C3/C2 ratio: < 0.2 (99.8th ptl); C3/Met ratio: < 0.43 (99.8th ptl). *Ptl* percentile. *CblA* methylmalonic acidemia CblA type, *CblB* methylmalonic acidemia CblB type, *CblC* methylmalonic acidemia with homocystinuria CblC type, *CblD* methylmalonic acidemia with homocystinuria CblD type, *CblE* methylmalonic acidemia with homocystinuria CblE type, *CblF* methylmalonic acidemia with homocystinuria CblF type, *CblG* homocystinuria CblG type, *CblJ* methylmalonic acidemia with homocystinuria CblJ type, *CblX* methylmalonic acidemia with homocystinuria CblX type, *CBS* cystathionine β-synthase deficiency, *C17* heptadecanoilcarnitine, *C2* acetylcarnitine, *C3* propionylcarnitine, *Hcys* homocysteine, *MCA* methylcitric acid, *Met* methionine, *MMA* methylmalonic acid, *MS* methionine synthase, *MTHFR* methylenetetrahydrofolate reductase, *MUT* methylmalonyl-CoA mutase deficiency, *NBS* newborn screening, *PA* propionic acidemia

At the end of 2014, we set up the second-tier test method above described. Cut-off values were chosen from our own experience, and the median age was 2,08 days with an interval of 2 to 7 days of age. The developed algorithm is shown in Fig. 1. When any of the primary markers is altered, samples undergo a second-tier test on the same DBS, which includes the simultaneous measurement of MMA, MCA and Hcys. The algorithm shows the classification of samples according to the concentration of second-tier tests in the first DBS. NBS is classified as normal, when all biomarkers are below the cut-off value; as doubtful, when one or more biomarkers show intermediate values and, as altered, when anyone of the biomarkers is clearly above the corresponding cut-off levels. In doubtful cases a second DBS is requested and primary as well as secondary biomarkers are again performed. In altered cases, newborns are referred to the clinical unit with the corresponding suspected diagnosis (Fig. 1).

To further classify the disease, laboratory testing in urine (MMA, MCA), plasma (total Hcys, MMA, acylcarnitines and aminoacids) and serum (vitamin B_12_) were assessed. Moreover, the same tests were performed in the mothers. In addition, folate was routinely measured when measuring vitamin B_12_ in the mothers.

Vitamin B_12_ deficient patients were treated with 1 mg intramuscular hydroxocobalamin and mothers were either treated or sent to their reference physician to be studied. Control analysis to the child was performed at two weeks post-treatment. The last control was performed at 6 months of age.

Information on feeding modalities, and short-term clinical outcome (presence/absence of clinical symptoms) was documented.

## Results

### Second-tier tests method validation

The optimized MS parameters in the MRM mode and the chromatographic separation of the compounds are shown in Additional file 4 and Additional file 5, respectively. The method was validated, and results are shown in Additional file 2.

### Screening results

**Fig. 2:**
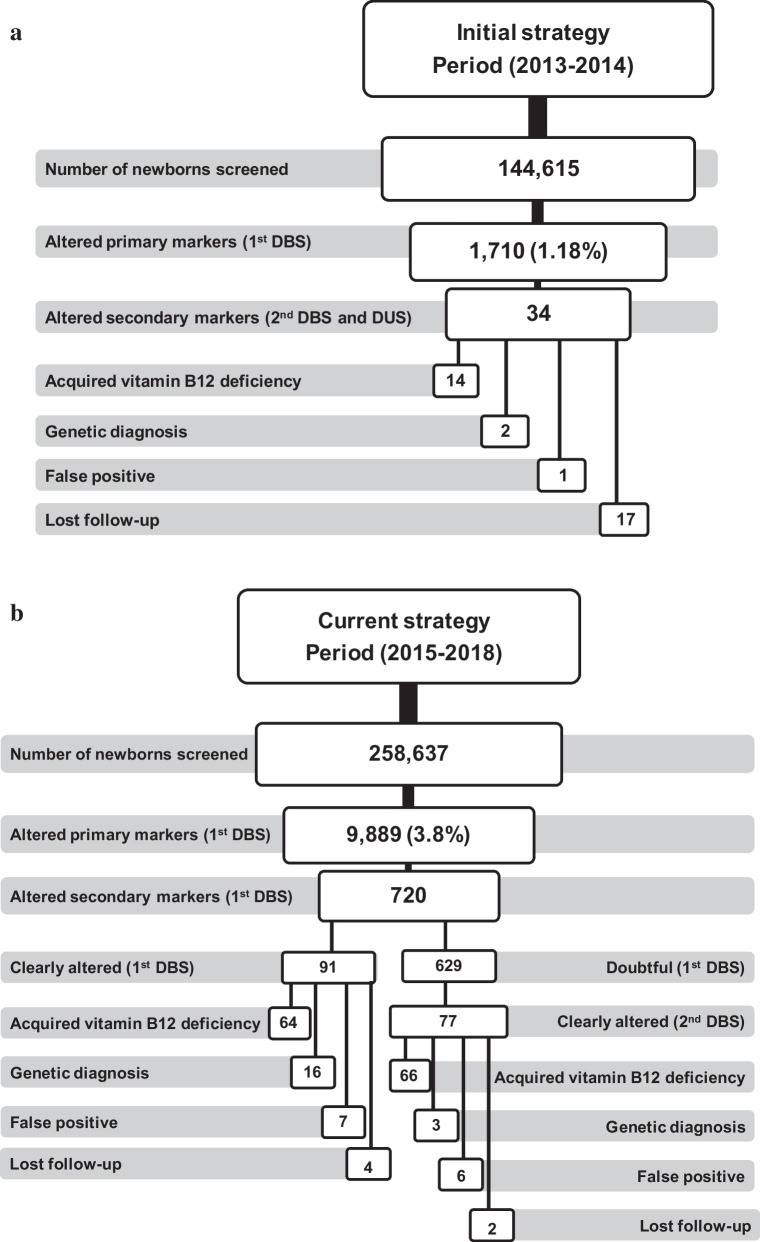
Results of newborn screening strategies. *DBS* dried blood spot, *DUS* dried urine spot

As mentioned above, when the second-tier tests were not available at our laboratory, our initial strategy was to ask a second DBS and DUS to the newborns with altered primary markers. In this period (2013–2014) a total of 144,615 newborns were screened, resulting in altered primary markers in 1710 (1.18%) (Fig. 2a), which disclosed a total of 34 cases with alterations, of which 14 were acquired vitamin B_12_ deficiencies, 2 genetic defects and 1 false positive. The follow-up was lost in 17 newborns. One false negative, diagnosed of methylmalonic acidemia with homocystinuria (CblC), was notified. The positive predictive value (PPV) for this strategy was 94%.

From 2015 to nowadays, second-tier tests (MMA, MCA and Hcys) were performed on the first DBS. The cut-off of C3 was lowered from 4.5 μmol/L (99th ptl) to 3.5 μmol/L (96th ptl) (Fig. 1). In the period 2015–2018 a total of 258,637 newborns were screened (Fig. 2b), of which 9,889 showed alterations of primary markers (3.8%), disclosing 720 newborns with altered secondary markers, of which 91 presented clearly altered values on the first DBS resulting in 64 acquired vitamin B_12_ deficiency, 16 were diagnosed with genetic defects and 7 were false positive. The follow-up was lost in 4 newborns. The remaining 629 were found to have intermediate or doubtful results, and according to our current algorithm, a second DBS was requested. Interestingly, 77 presented clearly altered values on the second DBS, disclosing 66 acquired vitamin B_12_ deficiency, 3 were diagnosed with genetic defects and 6 were false positive. The follow-up was lost in 2 newborns. In total, the current strategy resulted in 130 newborns with acquired vitamin B_12_ deficiency among 258,637 newborns screened (incidence 1:1989), 19 with genetic disorders (incidence 1:13,613) and 13 were false positive. No false negatives were notified. Using this strategy, the PPV was 93%.

As a part of the diagnostic work-up the genetic cause of the disease was established in 21 cases (2 cases of the initial strategy and 19 cases of the current strategy) with the following diagnoses: 4 methylmalonic acidemia with homocystinuria (3 CblC, 1 TCR defect), 7 isolated methylmalonicacidemia (1 CblA, 1 CblB and 5 MUT), 1 combined malonic and methylmalonic acidemia (ACSF3), 4 propionic acidemia, 1 SUCLA2 and, 4 homocystinuria (CBS deficiency). Details on mutations and follow-up of these patients are shown in Table 1. It is interesting to outline that among the Cbl C type patients, including the false negative of the first strategy, the clinical evolution seems not to be dependent on the mutations. It is also interesting to note that 4 out of 5 MUT patients remain asymptomatic. Propionic acidemia is the most severe disease as 3 out of 4 patients had symptoms before screening and two of them died at an early age. Fortunately, the follow-up of the 4 CBS patients showed that all remain asymptomatic.

**Table 1:** Characteristics and follow up of the patients with genetic diagnosis

Patient	Disease (type)	Mutation		Symptoms before or at screening	Follow-up
		Allele 1	Allele 2		
*Initial strategy*					
1	MMA (MUT)	c.977G > A	c.977G > A	no	Asymptomatic (7 y 5 m)
2	MMA + Hcys (CblC)	c.271dupA	c.271dupA	yes	Symptomatic (6y 8 m)
*Current strategy*					
3	MMA + Hcys (CblC)	c.566G > A	c.566G > A	no	Asymptomatic (6 y)
4	MMA + Hcys (CblC)	c.271dupA	c.271dupA	no	Asymptomatic (2y 9 m)
5	MMA (CblA)	c.594dupT	c.594dupT	yes	Asymptomatic (2y 8 m)
6	MMA (CblB)	c.260G > T	c.260G > T	yes	Symptomatic (6y)
7	MMA (MUT)	c.1415C > T	c.1190C > T	no	Asymptomatic (6y)
8	MMA (MUT)	c.655A > T	c.655A > T	yes	Symptomatic (5y 10 m)
9	MMA (MUT)	c.2159_2160delAT	c.2026G > A	no	Asymptomatic (4y)
10	MMA (MUT)	c.983T > C	c.2026G > A	no	Asymptomatic (3y 9 m)
11	Propionic acidemia (PCCA)	c.1409T > G	c.1409T > G	yes	Symptomatic (2y 11 m)
12	Propionic acidemia (PCCA)	c.1370G > A	c.1370G > A	yes	Symptomatic (exitus at 2,5 m)
13	Propionic acidemia	No mutations in PCCA or PCCB		no	Asymptomatic after liver transplant (5y 7 m)
14	Propionic acidemia (PCCB)	c.1218_1231delGGGCATCATCCGGCinsTAGAGCACAGGA	c.1218_1231delGGGCATCATCCGGCinsTAGAGCACAGGA	yes	Symptomatic (exitus at 12 d)
15	Hcys (CBS)	c.1039G > A	c.1330G > A	no	Asymptomatic (6y)
16	Hcys (CBS)	c.1039 + 5G > A	c.750G > C	no	Asymptomatic (4y 8 m)
17	Hcys (CBS)	c.1136G > A	c.1330G > A	no	Asymptomatic (4y 2 m)
18	Hcys (CBS)	c.572G > A	c.697T > G	no	Asymptomatic (2y 3 m)
19	MMA + malonic (ACSF3)	c.609T > A	c.1455C > T	yes	Symptomatic (4y 2 m)
20	MMA (SUCLA2)	c.1147C > T	Under study	no	Asymptomatic (3y 3 m)
21	MMA + Hcys (TCR)	c.256_258del	c.256_258del	no	Asymptomatic (3y 2 m)
*False negative (initial strategy)*					
22	MMA + Hcys (CblC)	c.271dupA	c.566G > A	yes	Asymptomatic 7y 7 m

*MMA* methylmalonic acidemia, *Hcys* homocystinuria, *CBS* Cystathionine Beta-Synthase, *TCR* transcobalamin receptor, *y* years, *m* months

**Fig. 3:**
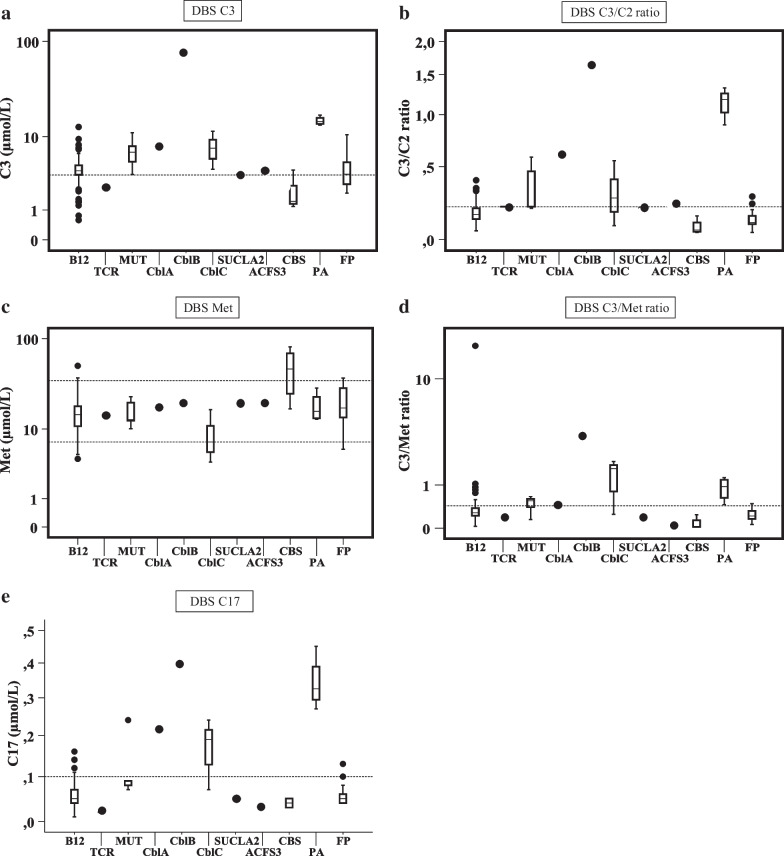
Altered values of primary markers on dried blood spots on selected newborns. Y-axis is represented in logarithmic scale. Values are depicted as box-and-whisker plots with minimum and maximum. Dashed lines represents the cut-off values in μmol/L: C3: initial strategy: > 4.5 (99th ptl); current strategy: > 3.5 (96th ptl); Met: 7–35 (1th ptl and 99.7th ptl, respectively); C17: > 0.1 (99.9th ptl); C3/C2 ratio: > 0.2 (99.8th ptl); C3/Met ratio: > 0.43 (99.8th ptl). Ptl: percentile. *ACSF3* combined malonic and methylmalonic acidemia due to acyl-CoA synthetase family, member 3 deficiency, *B12* vitamin B_12_ deficient newborns, *CblA* methylmalonic acidemia CblA type, *CblB* methylmalonic acidemia CblB type, *CblC* methylmalonic acidemia with homocystinuria CblC type, *CBS* cystathionine β-synthase deficiency, *C17* heptadecanoilcarnitine, *C2* acetylcarnitine, *C3* propionylcarnitine, *FP* false positive, *Met* methionine, *MUT* methylmalonyl-CoA mutase deficiency, *PA* propionic acidemia, *SUCLA2* beta-subunit of the ADP-forming succinyl-CoA synthetase deficiency, *TCR* transcobalamin receptor defect

Results of primary biomarkers on the first blood spot are shown in Table 2A. High C3 was found in a similar percentage both, for genetic or acquired conditions (80% and 81%, respectively). However, C3/C2, C3/Met and C17 were increased in a higher percentage in the group of genetic conditions. Low methionine was recorded in 2 genetic defects (CblC patients) and in 10 cases with acquired vitamin B_12_ deficiency. High methionine was only detected in 3 genetic cases (CBS deficient patients) and in 2 cases with acquired conditions. DBS results of primary biomarkers by individual groups are shown in Fig. 3; C3 and C3/C2 values were clearly above the cut-off values in MUT, CblA, CblB, CblC, and propionic acidemia while patients with SUCLA2 or ACSF3 defects showed values slightly above the cut-off for both biomarkers. Obviously, C3 and C3/C2 ratio were below cut-off in patients with CBS deficiency (Fig. 3a, b). High Met was characteristic of CBS deficiency whereas, moderate to low values for Met were found in several diseases (Fig. 3c). High values of C3/Met were found in the group of CblC patients and in one patient with acquired B_12_ deficiency (Fig. 3d); C17, used as an additional primary marker, was very high in propionic acidemia and in some conditions with methylmalonic acidemia (Fig. 3e).

**Table 2:** Altered biomarkers in genetic and acquired conditions

Biomarkers	Genetic conditions		Acquired conditions	
	N/total N	Percentage %	N/total N	Percentage %
*(A) Primary biomarkers*				
C3	17/21	80	117/144	81
C3/C2	14/21	67	32/144	22
C3/Met	12/21	57	24/144	17
C17	9/21	43	17/144	12
Low Met	2/21	9.5	10/144	7
High Met	3/21	14	2/144	1.4
(B) Second-tier test biomarkers*				
MMA	11/19	58	78/130	60
Hcys	9/19	47	113/130	87
MCA	10/19	53	6/130	5

*C3* propionylcarnitine, *C2* acetylcarnitine, *Met* Methionine, *C17* heptadecanoylcarnitine, *MMA* methylmalonic acid, *Hcys* homocysteine, *MCA* methylcitric acid, *N* number of diagnosed cases with altered biomarker, *total N* total number of diagnosed cases

**Fig. 4:**
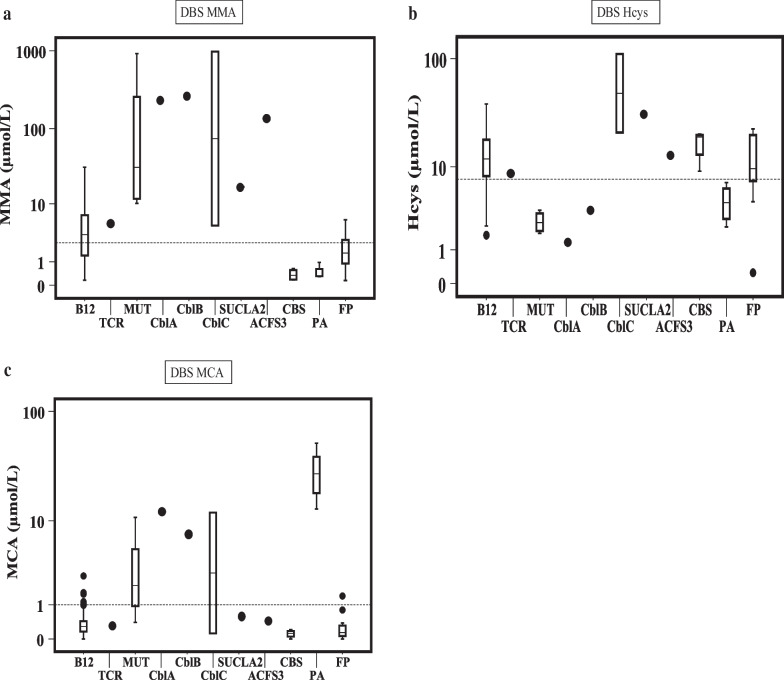
Second-tier test values on dried blood spots in newborns with altered primary markers. *Hcys* homocysteine, *MCA* methylcitric acid, *MMA* methylmalonic acid. Y-axis is represented as logarithmic scale. Values are depicted as box-and-whisker plots with minimum and maximum. Dashes lines represents the cut-off values (μmol/L) in the doubtful limit that implies the request of a second DBS: MMA > 2.5; Hcys > 7.5 and MCA > 1. *ACSF3* combined malonic and methylmalonic acidemia due to acyl-CoA synthetase family, member 3 deficiency, *B12* vitamin B_12_ deficiency, *CblA* methylmalonic acidemia CblA type, *CblB* methylmalonic acidemia CblB type, *CblC* methylmalonic acidemia with homocystinuria CblC type, *CBS* cystathionine β-synthase deficiency, *FP* false positive, *MUT* methylmalonyl-CoA mutase deficiency, *PA* propionic acidemia, *SUCLA2* beta-subunit of the ADP-forming succinyl-CoA synthetase deficiency, *TCR* transcobalamin receptor defect

Results of the second-tier test biomarkers are shown in Table 2B, the percentage of cases with MMA above the cut-off levels was very similar, both for genetic or acquired conditions (58% and 60% respectively), but genetic defects showed, in general, highest concentrations of this metabolite (Fig. 4a). Acquired conditions of vitamin B_12_ deficiency showed 87% of cases with high Hcys (Table 2B), and it was also high in all CblC and in 3 CBS patients but it was below the cut-off values in one CBS patient (9 µmol/L) (Fig. 4b). However, due to the high Met a second DBS was asked, resulting in a clearly high Hcys (68.2 µmol/L). Surprisingly, Hcys was also high in the patient with SUCLA2 defect (Fig. 4b), but it was due to a concomitant dietary deficiency of vitamin B_12_ in the mother and, consequently, in the newborn.

MCA was high in 53% of the genetic cases (very high in all propionic acidemias, and in some genetic defects) versus 5% of the acquired conditions (Table 2B, Fig. 4c).

### Vitamin B_12_ deficiency on the newborns

The diagnosis of vitamin B_12_ deficiency was established when vitamin B_12_ (in blood) was low (< 198 pmol/L) and, MMA (in blood or urine) and/or Hcys (in plasma) were elevated. This accounted for 11 patients of the initial strategy and 95 of the current strategy. In addition, 3 patients from the initial strategy and 35 patients from the current strategy were classified as functionally deficient. In these cases, MMA and Hcys were high, while for several reasons vitamin B_12_ could not be measured in some of the newborns.

### Vitamin B_12_ and Folate on the mothers

All mothers of deficient newborns, underwent vitamin B_12_ and folate measurement; 57 mothers had a very low vitamin B_12_ value (< 156 pmol/L), 47 had intermediate levels (156–222 pmol/L) and 25 had normal levels. Folate was high in 11 mothers. Interestingly, all of them showed low or very low plasma vitamin B_12_ concentration.

### Demographic data

Demographic data from vitamin B_12_ deficient cases were obtained from the newborn screening cards. Mother’s mean age was 30.5 years (range 18–43), of whom only 11 (7.6%) declare themselves as vegetarians. Regarding the mother’s nationality, 38.2% were Spanish, 31.3% Indian or Pakistani, 15.2% from Central and South America, 7.6% from North Africa (mainly Morocco), 4.2% from European countries, and 2.8% from Asia and the Middle East. Data was not available in one case (0.7%).

### Cost of the second tier test

Excluding personnel, the cost of test is 6 Euros, it was performed in 3,8% of the newborns resulting in an increase of 59.334 Euros in a total of 258.637 newborns screened. Therefore it results in an additional cost of 0,23 Euros per newborn.

## Discussion

The expanded NBS of Catalonia begun in 2013. Diseases included are shown in Additional file 3. Since propionic acidemia, methylmalonic acidemia and homocystinuria were included in our program and primary markers (C3, C3/C2 ratio, Met, C3/Met ratio or, C17) are somehow unspecific, second-tier tests were necessary to avoid false positive or false negative results. Our initial strategy for more than one year was to ask for a second DBS to reanalyze the primary markers and DUS to analyze organic acids (Fig. 2a), but the large number of samples requested for confirmatory testing precluded the long-term use of this strategy.

The setting up of the second-tier test for MMA, MCA and Hcys in a single step in the same DBS was established in our laboratory in 2015, which allowed us to decrease the cut-off values to avoid false negative results, without excessively increasing the number of false positive cases (Fig. 2b). Recently, a German pilot screening has demonstrated the usefulness of the second-tier test for the detection of vitamin B_12_ deficiencies [24]. These authors use two second-tier strategies of pathways involving vitamin B_12_ by measuring on the one hand tHcys, and on the other MMA, MCA ad 3-hidroxypropionate, while our strategy comprises a single test, which measures three metabolites together (Hcys, MMA and MCA). In addition, our procedure does not use any derivatization step, making the analysis simpler. However, a sensitive MS/MS, such as the one used in this study, is needed to accomplish these measurements.

Quantitative analysis in DBS for MMA, MCA and Hcys was established and validated with good results (Additional file 2). Interestingly, despite using different approaches, results obtained by Gramer et al. [24] were similar to ours, except for a higher incidence of acquired vitamin B_12_ deficiency in our population, 1:1,989 or 1:2,722 by excluding 35 newborns in which vitamin B_12_ was not measured, but with proven functional deficiency. The incidence reported by Gramer et al. [24] was 1:5,355 newborns, which is very close to that reported in Italy (1:5,000) [22] and far away from that reported in Minnesota with a detection rate of 3:100,000 newborns [20]. Recently, an incidence of 1:3,000 newborns in the Estonian screening program has been reported [23], while another recent German study of Munich NBS program revealed a much lower incidence of vitamin B_12_ deficiency [25]. Therefore, the incidence of this deficiency differs considerably among NBS programs and it might reflect different strategies used in each program, or even dietetic cultural factors dependent on the nationalities of origin, or other demographic factors. On the other hand, the Italian, Estonian and Municher programs [22, 23, 25] are mainly based on the use of C3 and MMA, while our program and the German pilot screening of Heidelberg [24] are based on the detection of metabolites of both cobalamin dependent pathways. In addition, the cut-offs used play a relevant role, as exemplified by our two strategies. When using the initial strategy (C3 cut-off: 4.5 µmol/L) the percentage of newborns with altered primary markers was 1.18%, while when using the current strategy (C3 cut-off: 3.5 µmol/L) the number increased to 3.8%, and decreased to 0.28% when applying the second-tier test in this sample (Fig. 2b). Consequently, the probability to pick-up both, acquired vitamin B_12_ deficient newborns as well as genetic alterations increases. Interestingly, the pilot study of Gramer et al. [24] speculated that the true incidence of vitamin B_12_ deficiency in their population might even be higher than it was found, as they only picked up moderate to severe cases of vitamin B_12_ deficiency. In fact, the results of our NBS program (using the current strategy) support their hypothesis. The higher incidence in our population was probably due to the lowest C3 cut-off, which allowed us to include not only moderate and severe cases but also mild cases. In addition, the incidence of genetic defects was also high, 1:13,612 newborns. Although it should be mentioned that these results included 3 deficiencies (SUCLA2, ACSF3 and TCR), which are not part of our primary panel and could be considered as incidental findings. In these cases, the diagnosis has been reached thanks to the genetic analysis through whole exome sequencing (WES). This methodology is cost-effective as there are 34 genes associated to these diseases. As a consequence, the incidental findings in our NBS program are not rare, but a secondary benefit of these findings is that early treatment can be started in some cases, as it is shown by TCR deficiency resulting in an asymptomatic individual at 3 years of age (Table 1), while in others it will avoid the diagnostic odyssey for the family and facilitate access to adequate genetic counseling [26, 27]. I would like to remark some main points, one is the high frequency of methylmalonic acidemia (MUT) and the modest frequency of methylmalonic acidemia with homcystinuria (CblC type) compared with other studies [28–30], another point is the good evolution of most patients after treatment and several years follow-up (15 out of 21 patients are at present asymptomatic). The worst condition is propionic acidemia, with only one asymptomatic patient, and the best is CBS deficiency being all patients asymptomatic after treatment. Similar results have been reported by Heumer M et al. [31].

Concerning biomarkers, our results showed C3 as the most unspecific marker, both for acquired and genetic defects, (Table 2A) and we agree with other authors [24, 32] that C3 could be not sensitive enough applying conventional cut-offs. It has been reported the ratio C3/C2 as more sensitive than C3 [21, 33]. In our hands, this ratio was more specific than C3, as it is more frequently high in genetic than in acquired conditions (67% versus 22% respectively, Table 2A). However, the sensitivity of a particular biomarker is dependent on the established cut-off [34]. In our population the cut-off for C3 was 3.5 μmol/L, that is quite low, with the purpose to avoid false negative results, as it has been previously described [35, 36]. This strategy, however, implies the analysis of second-tier tests to prevent an excessive number of false positives.

In agreement with Gramer et al. [24] Hcys is the best second-tier biomarker for the detection of acquired vitamin B_12_ deficiency (Table 2B) and could explain why other programs [20, 22, 25] have found a low number of vitamin B_12_ deficiencies since they do not use Hcys as second-tier test. In agreement with Hawthorne et al. reflections [37], the measurement of Hcys as second-tier test is an economical way to increase the number of vitamin B_12_ deficient infants identified, but also of some treatable genetic disorders.

Concerning MCA it has been proven to be a good marker for propionic acidemia as it was always very high in all detected cases. It was also high in some other genetic defects. However, it was found elevated only in few cases with acquired vitamin B_12_ deficiency (5%) (Table 2B). Consequently, MCA is very helpful to distinguish between both conditions.

Cases with acquired vitamin B_12_ deficiency were treated according to the established protocol, and thus avoiding the collateral damage associated to this condition. In addition, as a part of diagnostic work-up the genetic cause of the disease was established in 21 patients resulting in 15 asymptomatic individuals after several years of follow-up (Table 1). As a consequence, Screening of vitamin B12 deficiency has been incorporated in our screening program. In addition, the Autonomous Government of Catalonia recommended to avoid low B12 ingestion during pregnancy [38].

Regarding the hypothesis of Selhub et al. [39] on the adverse effects of folate supplementation on the metabolism of vitamin B_12_, we have not been able to establish a statistically significant relationship, as high serum folate and low vitamin B_12_ was only found in 11 mothers.

## Conclusions

The inclusion of MMA, MCA and Hcys as second-tier test in our NBS program was successful in detecting both, acquired vitamin B_12_ deficiency and genetic defects, with an incidence of 1:1.989 and 1:13,612 newborns, respectively.

Inclusion of second-tier test in our NBS program decreased drastically the recall rate due to false positive results of primary markers. On the other hand, it allowed us to decrease the cut-off of primary markers to avoid false negative results.

The best second-tier marker for acquired vitamin B_12_ deficiency was Hcys, and when MCA is high, it points to a genetic defect rather than acquired conditions. However, despite certain trends that point more to one condition than the other, it is not possible to distinguish between them in absolute terms. In these cases, the assessment of vitamin B_12_ will help to establish the differential diagnosis.

NBS programs including methylmalonic acidemia and homocystinuria should also include the screening of acquired vitamin B_12_ deficiency, since the benefits of its detection perfectly meet the criteria of Wilson and Jungner [40]. Therefore, screening for vitamin B_12_ deficiency has been incorporated in our screening program.

## Supplementary Information

**Additional file 1.** Additional material and methods.

**Additional file 2.** Validation results of methylmalonic acid, methylcitric acid and homocysteine on dried blood spots by UPLC-MS/MS.

**Additional file 3.** Diseases included in the neonatal screening program of Catalonia.

**Additional file 4.** Mass spectrometer parameters for the detection of methylmalonic acid, homocysteine, and methylcitric acid.

**Additional file 5.** MRM of the extracted ion chromatograms of methylmalonic acid homocysteine and methylcitric acid together with the corresponding deuterated compounds.

## Data Availability

Available on request.

## Abbreviations

ACSF3: Acyl-CoA synthetase family member 3
CblA: Methylmalonic acidemia CblA type
CblB: Methylmalonic acidemia CblB type
CblC: Methylmalonic acidemia with homocystinuria CblC type
CBS: Cystathionine β-synthase
C17: Heptadecanoylcarnitine
C2: Acetylcarnitine
C3: Propionylcarnitine
DBS: Dried blood spot
DUS: Dried urine spot
Hcys: Homocysteine
Hcys-_d8_: DL-homocysteine (3,3,3′,3′,4,4,4′,4′-_d8_)
MCA: Methylcitric acid
MCA-_d3_: 2-Methyl-_d3_-citric acid
Met: Methionine
MMA-_d3_: Isotopically labeled methylmalonic acid
MMA: Methylmalonic acid
MS/MS: Tandem mass spectrometry
MUT: Methylmalonyl-CoA mutase
NBS: Newborn screening
SUCLA2: Beta-subunit of the ADP-forming succinyl-CoA synthetase deficiency
TCR: Transcobalamin receptor

## References

[1] Therrell BL, Padilla CD, Loeber JG, Kneisser I, Saadallah A, Borrajo GJ (2015). Current status of newborn screening worldwide: 2015. Semin Perinatol.

[2] Garcia-Villoria J, Pajares S, López RM, Marin JL, Ribes A (2016). Neonatal Screening for Inherited Metabolic Diseases in 2016. Semin Pediatr Neurol.

[3] Fowler B, Leonard JV, Baumgartner MR (2008). Causes of and diagnostic approach to Methylmalonic acidurias. J Inherit Metab Dis.

[4] Huemer M, Kožich V, Rinaldo P, Baumgartner MR, Merinero B, Pasquini E (2015). Newborn screening for homocystinurias and Methylation disorders: systematic review and proposed guidelines. J Inherit Metab Dis.

[5] Baumgartner MR, Hörster F, Dionisi-Vici C, Haliloglu G, Karall D, Chapman KA (2014). Proposed guidelines for the diagnosis and management of methylmalonic and propionic acidemia. Orphanet J Rare Dis.

[6] Whitehead VM (2006). Acquired and inherited disorders of cobalamin and folate in children. Br J Haematol.

[7] Kuhne T, Rubl R, Baumgartner R (1991). Maternal vegan diet causing a serious infantile neurological disorder due to vitamin B12 deficiency. Eur J Pediatr.

[8] Graham SM, Arvela OM, Wise GA (1992). Long-term neurologic consequences of nutritional vitamin B12 deficiency in infants. J Pediatr.

[9] Rasmussen SA, Fernhoff PM, Scanlon KS (2001). Vitamin B12 deficiency in children and adolescents. J Pediatr.

[10] Weiss R, Fogelman Y, Bennett M (2004). Severe vitamin B12 deficiency in an infant associated with a maternal deficiency and a strict vegetarian diet. J Pediatr Hematol Oncol.

[11] KorenKe GC, Hunneman DH, Eber S, Hanefeld F (2004). Severe encephalopathy with epilepsy in an infant caused by subclinical maternal pernicious anaemia: case report and review of the literature. Eur J Pediatr.

[12] Jain R, Singh A, Mittal M, Talukdar B (2015). Vitamin B12 deficiency in children: a treatable cause of neurodevelopmental delay. J Child Neurol.

[13] Singh G, Le D, Schnabl K, Leaker MT, Steele M, Sparkes RL (2016). Vitamin B12 deficiency in infancy: the case for screening. Pediatr Blood Cancer.

[14] Malvagia S, Haynes CA, Grisotto L, Ombrone D, Funghini S, Moretti E (2015). Heptadecanoylcarnitine (C17) a novel candidate biomarker for newborn screening of propionic and Methylmalonic acidemias. Clin Chim Acta.

[15] La Marca G, Malvagia S, Pasquini E, Innocenti M, Donati MA, Zammarchi E (2007). Rapid 2nd-tier test for measurement of 3-OH-propionic and methylmalonic acids on dried blood spots: reducing the false positive rate for propionylcarnitine during expanded newborn screening by liquid chromatography-tandem mass spectrometry. Clin Chem.

[16] Matern D, Tortorelli S, Oglesbee D, Gavrilov D, Rinaldo P (2007). Reduction of the false-positive rate in newborn screening by implementation of MS/ MS-based second-tier tests: the Mayo Clinic experience (2004–2007). J Inherit Metab Dis.

[17] Monostori P, Klinke G, Richter S, Barath A, Fingerhut R, Baumgartner MR (2017). Simultaneous determination of 3-hydroxypropionic acid, methylmalonic acid and methylcitric acid in dried blood spots: second-tier LC-MS/MS assay for newborn screening of propionic acidemia, methylmalonic acidemias and combined remethylation disorders. PLoS ONE.

[18] Marble M, Copeland S, Khanfar N, Rosenblatt DS (2008). Neonatal vitamin B12 deficiency secondary to maternal subclinical pernicious anemia: identification by expanded newborn screening. J Pediatr.

[19] Hinton CF, Ojodu JA, Fernhoff PM, Rasmussen SA, Scanlon KS, Hannon WH (2010). Maternal and neonatal vitamin B12 deficiency detected through expanded newborn screening—United States, 2003–2007. J Pediatr.

[20] Sarafoglou K, Rodgers J, Hietala A, Matern D, Bentler K (2011). Expanded newborn screening for detection of vitamin B12 deficiency. JAMA.

[21] Yahyaoui R, Dayaldasani A, Rueda I, Blasco J, Serrano J, Navas VM (2012). Detection of vitamin B12 deficiency using expanded newborn screening in eastern Andalucia. Spain J Inherit Metab Dis.

[22] Scolamiero E, Villani GR, Ingenito L, Pecce R, Albano L, Caterino M (2014). Maternal vitamin B12 deficiency detected in expanded newborn screening. Clin Biochem.

[23] Reinson K, Kunnapas K, Kriisa A, Vals MA, Muru K, Ounap K (2018). High incidence of low vitamin B12 levels in Estonian newborns. Mol Genet Metab Rep.

[24] Gramer G, Fang-Hoffmann J, Feyh P, Klinke G, Monostori P, Mütze U (2020). Newborn screening for vitamin B12 deficiency in Germany—strategies, results, and public health implications. J Pediatr.

[25] Weiss KJ, Röschinger W, Blessing H, Lotz-Havla AS, Schiergens KA, Maier EM. Diagnostic challenges using a 2-tier strategy for methylmalonic acidurias: data from 1.2 million dried blood spots. Ann Nutr Metab. 2020;1–9. 10.1159/000508838 32683363

[26] Pajares S, López RM, Gort L, Argudo-Ramírez A, Marín JL, González de Aledo-Castillo JM (2020). An incidental finding in newborn screening leading to the diagnosis of a patient with *ECHS1* mutations. Mol Genet Metab.

[27] Yahyaoui R, Blasco-Alonso J, Gonzalo-Marín M, Benito C, Serrano-Nieto J, González-Gallego I (2020). Metabolic serendipities of expanded newborn screening. Genes.

[28] Nogueira C, Aiello C, Cerone R, Martins E, Caruso U, Moroni I, Rizzo C (2008). Spectrum of MMACHC mutations in Italian and Portuguese patients with combined methylmalonic aciduria and homocystinuria, cblC type. Mol Genet Metab.

[29] Richard E, Jorge-Finnigan A, Garcia-Villoria J, Merinero B, Desviat LR, Gort L (2009). Genetic and cellular studies of oxidative stress in methylmalonic aciduria (MMA) cobalamin deficiency type C (cblC) with homocystinuria (MMACHC). Hum Mutat.

[30] Yang N, Gong LF, Zhao JQ, Yang HH, Ma ZJ, Liu W (2020). Inborn errors of metabolism detectable by tandem mass spectrometry in Beijing. J Pediatr Endocrinol Metab.

[31] Huemer M, Kožich V, Rinaldo P, Baumgartner MR, Merinero B, Pasquini E, Blom HJ (2015). Newborn screening for homocystinurias and methylation disorders: systematic review and proposed guidelines. J Inherit Metab Dis.

[32] Campbell CD, Ganesh J, Ficicioglu C (2005). Two newborns with nutritional vitamin B12 deficiency: challenges in newborn screening for vitamin B12 deficiency. Haematologica..

[33] Armour CM, Brebner A, Watkins D, Geraghty MT, Chan A, Rosenblatt DS (2013). A patient with an inborn error of vitamin B12 Metabolism (cblF) detected by newborn screening newborn screening. Pediatrics.

[34] Estrella J, Wilcken B, Carpenter K, Bhattacharya K, Tchan M, Wiley V (2014). Expanded newborn screening in New South Wales: Missed cases. J Inherit Metab Dis.

[35] la Marca G, Malvagia S, Casetta B, Pasquini E, Donati MA, Zammarchi E (2008). Progress in expanded newborn screening for metabolic conditions by LC-MS/MS in Tuscany: update on methods to reduce false tests. J Inherit Metab Dis.

[36] Al-Dirbashi OY, McIntosh N, McRoberts C, Fisher L, Rashed MS, Makhseed N (2014). Analysis of methylcitrate in dried blood spots by liquid chromatography-tandem mass spectrometry. JIMD Rep.

[37] Hawthorne BS, Levy HL (2020). Can newborn screening for vitamin B_12_ deficiency be incorporated into all newborn screening programs?. J Pediatr.

[38] Pregnancy monitoring protocol in Catalonia. 3rd ed. Health Department. Government of Catalonia. Available from: https://scientiasalut.gencat.cat/bitstream/handle/11351/1204/protocol_seguiment_embaras_catalunya_2018.pdf?sequence=14.

[39] Selhub J, Morris MS, Jacques PF (2007). In vitamin B12 deficiency, higher serum folate is associated with increased total homocysteine and methylmalonic acid concentrations. Proc Natl Acad Sci USA.

[40] Wilson JMG, Jungner G and World Health Organization. Principles and practice of screening for disease. Geneva: WHO; 1968. Available from: https://apps.who.int/iris/handle/10665/37650.

