# Correction to: implementation of second-tier tests in newborn screening for the detection of vitamin B12 related acquired and genetic disorders: results on 258,637 newborns

**DOI:** 10.1186/s13023-023-02793-4

**Published:** 2023-07-13

**Authors:** Sonia Pajares, Jose Antonio Arranz, Aida Ormazabal, Mireia Del Toro, Ángeles García-Cazorla, Aleix Navarro-Sastre, Rosa María López, Silvia María Meavilla, Mariela Mercedes de los Santos, Camila García-Volpe, Jose Manuel González de Aledo-Castillo, Ana Argudo, Jose Luís Marín, Clara Carnicer, Rafael Artuch, Frederic Tort, Laura Gort, Rosa Fernández, Judit García-Villoria, Antonia Ribes

**Affiliations:** 1 Sección de Errores Congénitos del Metabolismo-IBC, Servicio de Bioquímica Y Genética Molecular, Hospital Clínic de Barcelona, C/ Mejía Lequerica S/N, Edificio Helios III, Barcelona, 08028 Spain; 2 Center for Biomedical Research Network on Rare Diseases (CIBERER), Madrid, Spain; 3 Unit of Metabolic Diseases, Hospital Vall D’Hebrón, Barcelona, Spain; 4 Inborn Errors of Metabolism Unit, Hospital Sant Joan de Déu, Barcelona, Spain; 5 Biomedical Research Institute, August Pi I Sunyer (IDIBAPS), Barcelona, Spain; 6 Maternal and Child Health Service, Public Health Agency of Catalonia, Health Department, Government of Catalonia, Barcelona, Spain

Following publication of the original article [1], we have been notified that the Funding note was published incorrectly. It should be as follows:

This research was supported, in part, by the Instituto de Salud Carlos III (PI19/01310) and co-funded by European Union (ERDF, “A way to make Europe”), the Centro de Investigación Biomédica en Red de Enferme dades Raras (CIBERER), an initiative of the Instituto de Salud Carlos III (Minis terio de Ciencia e Innovación, Spain). This study was also supported by the Agència de Gestió d’Ajuts Universitaris i de Recerca (AGAUR, 2017:SGR 1428) and the CERCA Programme/Generalitat de Catalunya.
