# Supplementary material for: Implementation of second-tier tests in newborn screening for the detection of vitamin B12 related acquired and genetic disorders: results on 258,637 newborns
Source: Orphanet J Rare Dis. 2021 Apr 30;16:195. doi: 10.1186/s13023-021-01784-7 (PMC8086297; doi:10.1186/s13023-021-01784-7)
Supplement: Supplementary file 1 — Additional file 1. Additional material and methods. [file 13023_2021_1784_MOESM1_ESM.pdf]

Additional file 1. Additional material and methods.

Subjects

403,252 newborns from the NBS program of Catalonia, between 2013 and 2018, were studied. All samples were collected between 48h and 7 days of life. Samples were stored at 4ºC until their analysis.

Aminoacids and acylcarnitines analysis

Aminoacids and acylcarnitines were analyzed by means of NeoBase™ Non-derivatized MS/MS kit (Perkin Elmer, Waltham, Massachusetts, USA) by tandem mass spectrometry, (Xevo TQD, Waters, Milford, MA, USA), as described by the manufacturer.

Organic acid analysis on dried urine spots (DUS)

Organic acids were analyzed as their trimethylsilyl derivatives (TMS) by gas chromatography-mass spectrometry (GC7890A / MS5975, Agilent Technologies, Santa Clara, CA, USA). The extraction procedure was performed according to Tanaka et al. [1] with some modifications. Briefly, 4 mL of saturated NaCl were added to a DUS of 3 x 5 cm and shaked for 30 min. After the addition of 25 µL of internal standard (undecanedioic acid 1.4 mmol/L) and 275 µL of HCl 4N, urine was extracted three times with 2 mL ethylacetate, the organic phases were collected in the same vial and evaporated under a gentle of nitrogen at room temperature. Derivatization with 90 µL of N,O-Bis (trimethylsilyl) trifluoroacetamide (BSTFA) at 60 ºC for 30 min was performed and 1µL was injected into the GC-MS.

[1] Tanaka K, West-Dull A, Hine DG, Lynn TB, Lowe T. Gas chromatography method of analysis for urinary organic acids. Description of the procedure and its application to the diagnosis of patients with organic acidurias. Clin Chem 1980; 26:1847-1853.
