# Supplementary material for: Implementation of second-tier tests in newborn screening for the detection of vitamin B12 related acquired and genetic disorders: results on 258,637 newborns
Source: Orphanet J Rare Dis. 2021 Apr 30;16:195. doi: 10.1186/s13023-021-01784-7 (PMC8086297; doi:10.1186/s13023-021-01784-7)
Supplement: Supplementary file 2 — Additional file 2. Validation results of methylmalonic acid, methylcitric acid and homocysteine on dried blood spots by UPLC-MS/MS. [file 13023_2021_1784_MOESM2_ESM.pdf]

Additional file 2. Validation results of methylmalonic acid (MMA), methylcitric acid (MCA) and homocysteine (Hcys) on dried blood spots by UPLC-MS/MS.

	**MMA**			**Hcys**			**MCA**		
**Calibration curve, n=5**									
Mean slope (range; SD).	0.0112 (0.0105-0.0126;0.0008)			0.0307 (0.0283-0.0324; 0.0018)			0.0261 (0.0193-0.0291;0.0040)		
Mean coefficient of linear regression, r^2 (range; SD).	0.9962 (0.989-0.9989;0.0040)			0.9907 (0.9842-0.9947;0.0039)			0.9965 (0.9933-0.9992;0.0025)		
**Within-day, n=10**									
	**Conc. analyzed (µmol/L)**	**Imprecision (% CV)**	**Accuracy (% RE)**	**Conc. analyzed (µmol/L)**	**Imprecision (% CV)**	**Accuracy (% RE)**	**Conc. analyzed (µmol/L)**	**Imprecision (% CV)**	**Accuracy (% RE)**
	3	7.9	-10.7	6	19.1	18.3	3	11.9	12.9
	15	6.3	-11.8	30	11.9	-7.3	15	6.3	-5.0
	40	5.9	-4.8	80	13.2	5.6	40	8.7	6.6
**Between-day, n=10**									
	**Conc. analyzed (µmol/L)**	**Imprecision (% CV)**	**Accuracy (% RE)**	**Conc. analyzed (µmol/L)**	**Imprecision (% CV)**	**Accuracy (% RE)**	**Conc. analyzed (µmol/L)**	**Imprecision (% CV)**	**Accuracy (% RE)**
	3	13.9	-4.22	6	20.8	-17.56	3	18.1	3.70
	15	9.9	-6.42	30	14.9	-10.37	15	11.5	-1.05
	40	10.2	0.96	80	14.9	-6.60	40	11.1	6.78
**Recovery (%), n=3**	**Conc. analyzed (µmol/L)**			**Conc. analyzed (µmol/L)**			**Conc. analyzed (µmol/L)**		
	3	26		6	74		3	33	
	15	77		30	94		15	85	
	40	98		80	118		40	109	
**LOD (µmol/L), n=3**	1			1			1		
**LOQ (µmol/L), n=3**	3			3			3		

Conc.: concentration; CV: coefficient of variation; Hcys: homocysteine; LOD: limit of detection; LOQ: limit of quantification; MCA: methylcitric acid; MMA: methylmalonic acid; n: number of replicates; RE: relative error; SD: standard deviation; UPLC-MS/MS: ultra performance liquid chromatography-tandem mass spectrometry.
