# Supplementary material for: Implementation of second-tier tests in newborn screening for the detection of vitamin B12 related acquired and genetic disorders: results on 258,637 newborns
Source: Orphanet J Rare Dis. 2021 Apr 30;16:195. doi: 10.1186/s13023-021-01784-7 (PMC8086297; doi:10.1186/s13023-021-01784-7)
Supplement: Supplementary file 3 — Additional file 3. Diseases included in the neonatal screening program of Catalonia. [file 13023_2021_1784_MOESM3_ESM.pdf]

Additional file 3. Diseases included in the neonatal screening program of Catalonia.

Inherited metabolic diseases

Phenylketonuria and Hyperphenyalaninemias

Maple syrup urine disease

Tyrosinemia type I

Citrulinemia

Homocystinuria (CBS deficiency)

Glutaric aciduria type I

Isovaleric acidemia

Methylmalonic aciduria

Methylmalonic aciduria with Homocystinuria

3-Hydroxy-3-methylglutaryl-CoA lyase deficiency

ß-Ketothiolase deficiency

Propionic acidemia

Medium chain acyl-CoA dehydrogenase deficiency

Very long chain acyl-CoA dehydrogenase deficiency

Mitochondrial trifunctional protein deficiency

Multiple acyl-CoA dehydrogenase deficiency

Primary carnitine deficiency

Carnitine palmitoyltransferase 1 deficiency

Carnitine palmitoyltransferase 2 deficiency

Carnitine-acylcarnitine translocase deficiency

Other diseases

Cystic Fibrosis

Congenital Hypothyroidism

Sickle cell disease

Severe combined immunodeficiency
