# Supplementary material for: Implementation of second-tier tests in newborn screening for the detection of vitamin B12 related acquired and genetic disorders: results on 258,637 newborns
Source: Orphanet J Rare Dis. 2021 Apr 30;16:195. doi: 10.1186/s13023-021-01784-7 (PMC8086297; doi:10.1186/s13023-021-01784-7)
Supplement: Supplementary file 4 — Additional file 4. Mass spectrometer parameters for the detection of methylmalonic acid, homocysteine, and methylcitric acid. [file 13023_2021_1784_MOESM4_ESM.pdf]

Additional file 4. Mass spectrometer parameters for the detection of methylmalonic acid, homocysteine, and methylcitric acid.

Analyte	Cone voltage (V)	Collision energy (eV)	MRM transition	Retention time (min)
MMA	15	10	117>73	0.89
MMA-$_{d3}$	15	10	120>76	0.89
Hcys	60	15	136>90	0.57
Hcys-$_{d8}$	60	15	140>94	0.57
MCA	30	13	205>125	0.95
MCA-$_{d3}$	30	13	208>128	0.95

Hcys: homocysteine; Hcys-$_{d8}$: homocysteine (3,3,3',3',4,4,4',4'-$_{d8}$); MCA: methylcitric acid; MCA-$_{d3}$: MCA-$_{d3}$: 2-methyl-$_{d3}$-citric acid; MMA: methylmalonic acid; MMA-$_{d3}$: isotopically labeled methylmalonic acid; MRM: multiple reaction monitoring.
