# Supplementary material for: Implementation of second-tier tests in newborn screening for the detection of vitamin B12 related acquired and genetic disorders: results on 258,637 newborns
Source: Orphanet J Rare Dis. 2021 Apr 30;16:195. doi: 10.1186/s13023-021-01784-7 (PMC8086297; doi:10.1186/s13023-021-01784-7)
Supplement: Supplementary file 5 — Additional file 5. MRM of the extracted ion chromatograms of methylmalonic acid homocysteine and methylcitric acid together with the corresponding deuterated compounds. [file 13023_2021_1784_MOESM5_ESM.pdf]

Additional file 5. MRM of the extracted ion chromatograms of methylmalonic acid homocysteine and methylcitric acid together with the corresponding deuterated compounds.

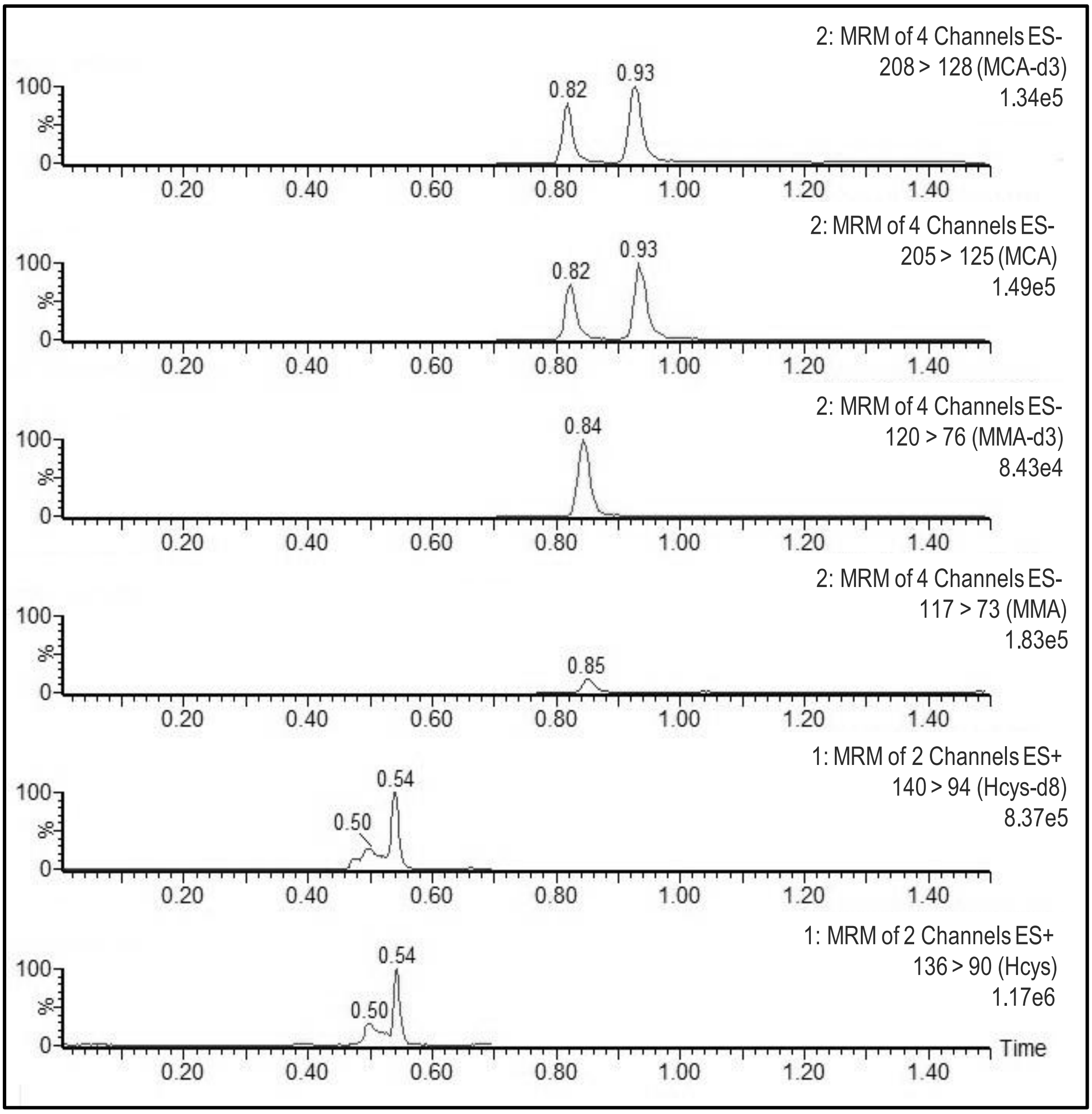

Hcys: homocysteine; Hcys-$_{d8}$: homocysteine (3,3,3',3',4,4,4',4'-$_{d8}$); MCA: methylcitric acid; MCA-$_{d3}$: MCA-$_{d3}$: 2-methyl-$_{d3}$-citric acid; MMA: methylmalonic acid; MMA-$_{d3}$: isotopically labeled methylmalonic acid; MRM: multiple reaction monitoring.
